# COVID-19 lockdown controls and human rights abuses: the social marketing implications

**DOI:** 10.35241/emeraldopenres.13810.1

**Published:** 2020-07-17

**Authors:** Ben Odigbo, Felix Eze, Rose Odigbo

**Affiliations:** 1 Department of Marketing, University of Calabar, Calabar, Cross River, 540242, Nigeria; 2 Department of Marketing/Entrepreneurship Development Centre, University of Calabar, Calabar, 540242, Nigeria; 3 Department of Public Law, Faculty of Law, University of Nigeria Nsukka, Enugu, 500001, Nigeria

**Keywords:** Covid-19 Lockdown, Covid-19 Curfews, Human Rights Abuses, International Conventions on Human Rights, Social Marketing.

## Abstract

**Background: **This work is a situation analysis of reported human rights abuses that have characterized the COVID-19 controls and lockdown in some countries of the world. This is as documented by reliable mass media sources, relevant international organizations and human rights non-governmental organizations between January 2020 to April 2020.

**Methods: **A combined content analysis, critical analysis, and doctrinal method is applied in this study in line with the reproducible research process. It is a secondary-data-based situation analysis study, conducted through a qualitative research approach.

**Findings: **The findings revealed among other things that: COVID-19 lockdowns and curfews’ enforcement by law enforcement officers contravened some peoples’ fundamental human rights within the first month. Security forces employed overt and immoderate forces to implement the orders. The lockdown and curfew enforcements were not significantly respectful of human life and human dignity. The COVID-19 emergency declarations in some countries were discriminatory against minorities and vulnerable groups in some countries.

**Research limitations/implications: **This report is based on data from investigative journalism and opinions of the United Nations and international human rights organizations, and not on police investigations or reports. The implication of the study is that if social marketing orientations and risk communication and community engagement attitudes were given to the law enforcement officers implementing the COVID-19 lockdowns and or curfews, the human rights and humanitarian rights breaches witnessed would have been avoided or drastically minimized.

**Originality: **The originality of this review is that it is the first to undertake a situation analysis of the COVID-19 lockdowns and curfews human rights abuses in some countries. The study portrayed the poor level of social marketing orientations and risk communication and community engagement attitudes amongst law enforcement officers, culminating in the frosty police-public relationships.

## Introduction

The World Health Organization ( WHO, 2020), seeing the alarming severity of coronavirus spread in many countries, on March 11, 2020, announced it is now a global pandemic. The new Coronavirus (COVID-19), was first reported in Wuhan, China, in December 2019. Since then, it has become a huge threat to lives, economic and social wellbeing of all citizens of the world. Mindful of this global emergency, the WHO (2020), urged governments all over the world to take urgent and concerted steps towards arresting the spread. In response to this onerous call, many governments of the world have initiated actions aimed at preventing or containing this rampaging enemy from exterminating their citizens, but in a fashion considered to be more on the side of human rights breaches and high-handedness ( The Guardian, 2020a). According to Amnesty International (2020b), during the 2019–20 coronavirus pandemic, human rights violations including censorship, discrimination, arbitrary detention, xenophobia have been reported from different parts of the world. The reported human rights violations pose serious hindrances to effective handling of public health emergencies ( Amnesty International, 2020b). The World Health Organization added its voice that stay-at-home measures for slowing down the pandemic must not be done in such a way as to jeopardize peoples’ human rights ( WHO, 2020).

The United Nations (1948) Universal Declaration of Human Rights (UDHR) has provisions for quality and equitable life for the people of all nations. Article 1 of the Convention recognizes the freedom, equality, dignity and rights of all human beings. That people should, therefore, act towards one another in a spirit of brotherhood. The Convention’s Article 3 adds that all the people of the world must be allowed their rights to life, liberty and security everywhere. Article 5 of the Convention protects the citizens of the world against torture, cruelty, inhumanity, dehumanizing treatments or punishments. Article 9 of the Convention is meant to protect the citizens of the world against arbitrary arrests, detentions or punitive banishments from one’s country. These UDHR provisions are also *in pari materia* with certain provisions of many countries’ Constitutions ( Sections 33(1), Sections 34(1), Sections 41(1), and Sections 305(1, 3a, 3b, 3c) of the 1999 Nigerian Constitution), as amended.

Even though, the Siracusa Principles on states of emergency and freedom of movement, which is recognized by the United Nations Economic and Social Council, and the United Nations Human Rights Commission, allows some level of governmental restrictions on human rights for reasons, during public health or national emergencies, but this must be done in a lawful, inevitable, and commensurate manner ( International Commission of Jurists, 1985). Again, the International Covenant on Economic, Social and Cultural Rights, adopted by almost all countries in the world, provides that everyone has the right to attain highest standards in health, be it emotional, physical or mental ( OHCHR, 2020).

Therefore, even though it is one of the social duties of governments to ensure the prevention, and management of epidemic and endemic diseases in any country ( International Commission of Jurists, 1985), this must be in consonance with citizens’ rights to health ( OHCHR, 1976), which agrees with provisions of the International Bill of Rights (IBR). The IBR enshrines peoples’ rights to all areas of human needs: physiologically, social, security, education, dignity, life, non-discrimination, equality, prohibition against abuses, privacy, access to information, and legitimate freedoms of all kinds, says the Washington Organization for Latin Ameria ( WOLA, 2020). States of emergencies should also consider and accommodate the peculiar situations and needs of the physically challenged, disadvantaged populations or marginalized groups ( International Commission of Jurists, 1985; OHCHR, 2020; UNHCR, 1976).

Annoyed with the rate of human rights abuses being reported across the globe, the UN High Commissioner for Human Rights, Michelle Bachelet, warned that countries and governments must be reminded that emergency responses to the coronavirus must respect peoples’ fundamental human rights ( Aljazeera, 2020b; OHCHR, 2020).

Social marketing experts believe that all these problems could have been addressed through pre-programme public enlightenment mass education campaigns on what COVID-19 is all about, the inherent dangers to individuals and society and the beneficial preventive measures expected from everyone. According to the World Health Organization ( WHO, 2020), this could be achieved by employing risk communications and community engagement tools at all levels of the society. Meanwhile, social marketing concept has been used globally to tackle various health challenges ranging from maternal and child health, risky behavior (smoking), campaign on tuberculosis, female genital mutilation (FGM), anti-alcoholism, anti-drug, and many more ( Odigbo *et al.*, 2018). The essence of social marketing is to use a combination of 4Ps marketing-mix variables (product, price, promotion and place), and another 4Ps, public relations-cum-managerial variables (publics, policy, partnership and purse-string), to induce people to understand and willingly accept a social course, for their own benefit and the benefit of society, and mankind in general in the context of COVID-19.

## Literature review

### Covid-19 lockdown human rights abuse and international conventions

Reports indicate that many countries are implementing the COVID-19 lockdown with significant cases of human rights abuses. For instance, just two weeks after the imposition of lockdown, security forces enforcing it killed 18 Nigerians, while COVID-19 killed 12 persons within the same period ( Africanews, 2020; Aljazeera, 2020a; Amnesty International, 2020a; BBC, 2020a; Human Rights Watch, 2020a; Idris, 2020; Khalid, 2020; Nigeriarights, 2020; Ojukwu, 2020). It was reported that six people lost their lives in Kenya due to police brutality within the first 10 days of COVID-19 dusk-to-dawn curfew in that country ( Human Rights Watch, 2020a). In Philippines, the lockdown measures were reportedly characterized by police brutality, abuses and massive prison deaths arising from overcrowding due to the coronavirus ( Alindogan, 2020; Aspinwall, 2020; The Diplomat, 2020c), so also with Iran ( Human Rights Watch, 2020j). In Sri Lanka the lockdown was allegedly used to implement press censorship and curtail public free expression ( Ganguly, 2020; Human Rights Watch, 2020e).

In the Central Asia region countries of Uzbekistan, Turkmenistan and Kyrgyzstan, reports have it that there were abuse of human rights in governments’ responses to the Covid-19 pandemic. These came in form of limited access to information on the levels of spread of the virus in those countries, and imposition of restrictions on the reportage by anyone ( Human Rights Watch, 2020d). Some of the governments used it to clampdown on journalists, perceived enemies, health personnel, social activists, and also implemented quarantine measures in harsh and arbitrary ways ( Alindogan, 2020; Aljazeera, 2020b). Other governments hiding under COVID-19 lockdown, used it to suppress activists’ and journalists’ freedom of expression rights ( Amnesty International, 2020b; Aspinwall, 2020; DailyMail, 2020; Ganguly, 2020; Human Rights Watch, 2020d; The Diplomat, 2020a). In other countries, the COVID-19 lockdown implementation has been turned into a killing spree by trigger-happy law enforcement officers ( Human Rights Watch, 2020d). Yet in some countries, vulnerable populations, like women and children, face increased risk of abuses, domestic violence, sexual abuse and rape during the lockdowns ( Al-monitor, 2020; The Guardian, 2020a), while prisoners in some countries are exposed to dire threats to their lives ( Human Rights Watch, 2020d).

The UN has warned tacitly that some countries are flouting peoples’ human rights in the guise of checkmating coronavirus spread, and called this “a human rights disaster” ( OHCHR, 2020). According to the UN High Commissioner for Human Rights, Michelle Bachelet, such countries should desist from infringing on peoples’ fundamental rights in the implementation of their emergency measures. This warning was followed by a UN report which mentioned 15 countries where allegations of COVID-19 lockdown enforcement human rights abuses were rampant ( Human Rights Watch, 2020f; OHCHR, 2020). The countries said the report include: Nigeria, Kenya, South Africa, the Philippines, Sri Lanka, El Salvador, the Dominican Republic, Peru, Honduras, Jordan, Morocco, Cambodia, Uzbekistan, Iran and Hungary. The UN highlighted that in some of the listed countries, security agencies used excessively crude force to ensure compliance to lockdowns and curfews ( Human Rights Watch, 2020f; The Diplomat, 2020c).

The International Covenant on Civil and Political Rights ( ICCPR, 1976a), one of the international human rights laws, states that when restrictions on rights are occasioned by public health or national emergency concerns, it must be implemented in a lawful, inevitable, and commensurate manner. Under this, people are guaranteed their rights to health, even to the highest possible standards. This places on governments, obligations to ensure the prevention of diseases and to provide medical care to the public.

Article 4 of the ICCPR human rights law also says that in the event of serious public health emergencies, stepping down on some rights may be justifiable, only when they are strictly inevitable, but must not be arbitrary or discriminatory in the implementation, and of limited timeframe. It must also respect human dignity, open to review, and targeted at achieving the set health objective(s), and have a legal basis. ICCPR (1976b) in Article 7 decries torture, cruelty, inhumanity or demeaning treatments or punishments on anyone. Article 9 (1) of the ICCPR states that people must be given their rights to liberty and security of persons, and that no person shall be deprived of this right, except strictly within the ambits of established rule of law.

Hence, States were therefore advised to adhere to human rights-based approaches in their COVID-19 pandemic controls, in order not to abridge rule of laws and achievement of healthy societies where everyone’s human rights are protected ( Bachelet, 2020; OHCHR, 2020). Based on the foregoing, therefore, the following research questions are drawn for this study:

RQ1: Was there a significant violation of fundamental human rights in the covid-19 lockdowns and curfews implementations within the first one month?

RQ2: Did security forces in the countries under review use excessive force to enforce lockdowns and curfew?

RQ3: Were the lockdowns and curfew enforcements significantly lawful, necessary and proportionate?

RQ4: Were all the citizens of the countries under review guaranteed their rights to the highest possible quality health standards?

RQ5: Were the lockdowns and curfew enforcements significantly respectful of human life and human dignity?

RQ6: Were emergency declarations based on the Covid-19 outbreak significantly used to discriminate against some groups or individuals?

### COVID-19 lockdown and social marketing

Social marketing could be used to achieve the willing acceptance, cooperation and support of a populace towards the COVID-19 lockdown and curfews. This has been proved true in many health communications’ and intervention studies ( Odigbo, 2016; Oti *et al.*, 2016). Kotler & Zaltman (1971), says that social marketing involves the designing, implementing and controling of social programs in order to influence their acceptability by target publics. It includes the marketing of social ideas through product designing, pricing, communication, distribution and research. Therefore, social marketing tools are used to change the negative or undesired attitudes and behavior of a target populace in the desired positive direction ( Hastings & McDermott, 2006; Kotler & Zaltman, 1971). So, social marketing encourages people to adopt social behavior that will be beneficial to all in society ( Kotler & Roberto, 1989; Ricordeau *et al.,* 2003).

Social marketing has been successfully used in the health areas like HIV/AIDS campaigns, to achieve desired behavior changes amongst the populace ( Odigbo *et al.*, 2017). Social marketing campaigns have also been used as a financial costs reduction tool in the health arena in some countries ( Oti *et al.*, 2016). For instance, social marketing has been used to improve maternal and child health, leading to increased patronage of health care centers, reduced complicated deliveries, improved newborn cares, and increased vaccination with consequent decline in medical bills, maternal and infant mortality rates ( Seetharam *et al.*, 2014). Hence, social marketing could also be used for effective public enlightenment ( Odigbo *et al.*, 2016), on the dangers of the coronavirus, the preventive measures against it, the proper things to do when contracted, the “dos and don’ts” over it, and the truths regarding the myths and rumors ( WHO, 2020), so as to achieve a smooth public acceptance of the lockdown enforcements by law enforcement officers, and forestall the ugly civilian-police confrontations that have culminated into avoidable deaths in some countries.

### COVID-19 lockdown and the 8Ps of social marketing

 The 8Ps of social marketing-mix elements that could be deployed in effective implementation of health communications ( Odigbo, 2016), therefore, also for the covid-19 lockdown and curfew enforcements include: the *product* (e.g. COVID-19 personal protective equipment, vaccines, gloves, face masks, sanitizers, etc.); the *price* (e.g., going to health centers, receiving a COVID-19 test, staying in isolation centers, social-distancing from friends and well-wishers, even family members in extreme cases, obeying stay-at-home orders, complying with law enforcement officers, government and health personnel controlling the covid-19, willing acceptance of business closures, job stoppages, and other deprivations); the *place* (different testing locations, hospitals, health centers, residential areas, etc.); the *promotion* (hand-washing, social-distancing, coronavirus test, anti-COVID-19 behavior ethics, and cooperation with governments, law enforcement agents and healthcare personnel); *the policy* (e.g., WHO regulations, governmental regulations and laws, international cooperation with other countries); *purse strings* (internal and external funding); and *partnership* (global cooperation with the World Health Organization, other countries and international organizations). A careful implementation of all these will bring about the willing cooperation and positive behavioral changes and attitudes of the populace towards COVID-19 lockdowns and curfew implementation.

### Origin and spread of COVID-19

COVID-19 was reported first in Wuhan, China, in December 2019. It advanced quickly all over the thickly-populated country ( CDC, 2020), to Hubei between December 2019 and February, 2020. It has gotten to 1,386 counties across all the 31 provinces. Today, coronavirus has spread like wild fire to all parts of the world. As at June 26, 2020, 213 countries and territories around the world have the coronavirus, with 9,753,786 reported cases, and 492,652 deaths globally ( Worldometer, 2020).

In January 30, 2020, the Coronavirus was declared a global public health emergency, by the World Health Organization (WHO), mindful of its devastating effects in many countries. WHO declared COVID-19 a pandemic, meaning a global epidemic or disease that has affected all nations of the world, on March 11, 2020 ( WHO, 2020).

Today, no country is spared of COVID-19, and all countries are coming up with novel drastic measures to checkmate the spread among their citizens. These include border closures, suspension of flights, partial or total lockdowns and curfews, with unbearable consequences on their citizens. For instance, in the United Kingdom, all schools have been closed in Scotland, Wales and Northern Ireland as measures to prevent the COVID-19 ( The Guardian, 2020a). In the United States, President Trump compares the sacrifices needed to curb COVID-19 as akin to those made during World War II, to what he called “the invisible enemy” ( Apnews, 2020). In Canada, a state of emergency has been declared in Ontario, which the Ontario’s Premier, Doug Ford, said was because Covid-19 constitutes a grave danger of unimaginable quantum, which must be dealt with decisively without any delay ( The Guardian, 2020a).

The government of Belgium also announced its first lockdown from March 18 to April 5, saying the citizens will only leave their homes for essential shopping for food, drugs and bank transactions in cases of emergencies ( Newstrust, 2020). In Austria, large gatherings, schools and shop owners selling non-essential goods were banned from opening ( Newstrust, 2020). Many countries imposed travel bans to curb the COVID-19 ( The Economist, 2020). Other countries shut their borders and international airports to travelers into or outside the countries ( Aljazeera, 2020b; NewYork Times, 2020). Corporate organizations around the world also closed up ( Jarvis, 2020; Traveldailynews, 2020). There is a spirited effort all over the world to discover vaccines or cure for the COVID-19 ( Andersen *et al.*, 2020). A UNESCO report says that over 160 countries closed all educational institutions nationwide, which affected over 87% of students globally, ( UNESCO, 2020). The International Monetary Fund ( IMF, 2020) has announced that COVID-19 has driven the world into another global recession, of probably more devastating impact than the 2008 global financial crisis ( Havard Business Review, 2020). Even though the World Health Organization says that COVID-19 has a zoonotic origin, that is emerged from an animal source, and that it has no evidence it originated from a Chinese lab ( WHO, 2020), the US President, Donald Trump, says that China has to be investigated on the origin of COVID-19, especially its scientific lab in Wuhan ( BBC, 2020b; CNN, 2020; The Guardian, 2020b). The U.K. Defense Minister, Ben Wallace, also accused China over the coronavirus outbreak ( CNBC, 2020). However, scientists and virologists have debunked this conspiracy theory and laboratory origin of COVID-19. This include a group of 27 prominent public health scientists cutting across countries, in an online publication by The Lancet released on Feb. 18, 2020 ( Cohen, 2020).

## Methods

A combined content analysis, critical analysis and doctrinal method is employed in this study, through a qualitative and quantitative approach. A Boolean search of mass media reports related to terms of COVID-19 was done. The exact search terms used were: “COVID-19 lockdown”, “COVID-19 curfews”, “COVID-19 human rights abuses”, “Human Rights Law”, “International Human Rights’ Conventions”, and “Universal Declaration of Human Rights”. Searchers were conducted between May 13 and May 28, 2020, mainly of COVID-19 lockdown/curfew management human rights issues that occurred within the first three weeks in many countries. The search engines employed were Google and Yahoo, since we are concerned mainly with mass media articles. The last search date was May 28, 2020. Since there were limited published articles on COVID-19 then, the sources of information for the analysis were mass media articles, and international instruments, covenants and or conventions on human rights.

The eligibility criteria used included articles from recognized national and or international mass media sources will be included in the study, and the opinion of international authorities on health and human rights (e.g., the World Health Organization, the UN Universal Declaration of Human Rights ( UDHR, 1948), the UN High Commission on Human Rights, International Covenant on Civil and Political Rights ( ICCPR, 1976b), International Commission of Jurists (1985), Human Rights Watch, Amnesty International, and national/international human rights organizations. Reports from local mass media sources that are not internationally recognized were excluded. The variables and data extracted for the analysis were mainly reports of human rights’ abuses that occurred within the period under review, due to COVID-19 lockdowns/curfews enforcement.

A five-step secondary data research process is adopted ( Denyer & Tranfield, 2009), to study issues related to COVID-19 lockdowns/curfews and human rights abuses in this study. The first step was by clearly defining the search criteria and the time period during which the COVID-19 lockdowns/curfews and human rights abuses occurred, and the consequent specific research questions and scope.

The second step was a sourcing of the relevant literature by selecting the following words or phrases that appeared in the mass media, statutes and conventions: “COVID-19 Lockdown”, “COVID-19 Curfews”, “Human Right Abuses”, “Human Right Laws,” and “International Conventions on Human Right.” A UN report mentioned 15 countries where the allegations of COVID-19 lockdown enforcement human rights abuses were rampant: Nigeria, Kenya, South Africa, the Philippines, Sri Lanka, El Salvador, the Dominican Republic, Peru, Honduras, Jordan, Morocco, Cambodia, Uzbekistan, Iran and Hungary ( Bachelet, 2020; OHCHR, 2020). The sample size for this study is comprised of a selection of 10 countries (out of the 15 listed countries in that UN OHCHR report). Another two countries where law enforcement agencies are reportedly used immoderate and sometimes fatal force to ensure lockdowns and curfews’ compliance is added to the 10, making a total sample of 12 countries. The 12 selected sample countries are: the Dominican Republic, El Salvador, Iran, Kenya, Morocco, Nigeria, the Philippines, South Africa, Sri Lanka, Uzbekistan, Kazakhstan and Kyrgyzstan, as displayed in Table 1.

**Table 1.** An overview of COVID-19 lockdown and curfews human rights abuses in the selected countries.

S/N	Country	Types of COVID-19 Lockdown / Curfew Human Rights Abuses	Reported By	Duration
1	Nigeria	18 persons killed. 33 tortured, inhumane and degrading treatments, unlawful arrests and detentions, seizure/confiscation of properties, Sexual molestations, Brutalities, Bribery.	United Nations, Amnesty International, NHRC, HRW, Africanews.com, BBC news, Khalid, Ishaq, Aljazeera, Tony Ojukwu. Nigeriarights.gov. Local media.	Between March 30 to April 15 (15 Days)
2	Kenya	6 cases of deaths linked to police, police violence, Brutalities, Extortions, unlawful arrests and detentions, Teargassing people. Excessive force.	United Nations, Aljazeera, Human Rights Watch. Local media.	March 27 to April 22 (26 Days)
3	South Africa	shootings, beatings, teargassing, and water bombing people, sexual abuse and exploitation. Reported *gender-based violence during lockdown.*	HRW, Human Rights Commission of South African, Local media.	March 27, 2020 to March 31, 2020. (4 Days).
4	Philippines	120,000 arrests, Deaths in prison, Use of Excessive force, Police violence and brutalities, Demolition of informal settlements. "Shoot to kill orders." Denial of social distancing to prisoners.	United Nations, Aljazeera, HRW, Jamela Alindogan, The Diplomat, Aspinwall Nick,	Between March 15 to April 03, (19 Days)
5	Sri Lanka	10,039 Police arrests, Suppression of freedom of expression, 2,489 vehicles seized, Blanket news censorship, security force abuses.	HRW, News.Ik, *Meenakshi Ganguly,*	20th of March to April 03. (13 days)
6	El Salvador	Arbitrary arrests, arbitrary detention of 1,200 people, poor access to health services, excessive use of force. Police violence and brutalities. “The use of lethal force.” 77 people were murdered in prisons. Denial of social distancing to prisoners.	Human Rights Watch, WOLA, Local media. Miamiherald	Between April 24 and 27, 2020.
7	Dominican Republic	Police arrested 48,000 people, Slaps a Doctor, Arbitrary arrests, arbitrary detention. Use of force.	Dailymail. *Amnesty International.* Aljazeera. OHCHR.	Mar 23, 2020 to 25 April 2020
8	Uzbekistan	Arbitrary restrictions on Journalists and activists. Suppressing freedom of expression. Raids, Confiscation of vehicles.	Human Rights Watch (April 23, 2020). The Diplomat.	Mar 23, 2020 to April 23.
9	Kyrgyzstan	Denial of pass permits to lawyers and journalists. Arrest of more than 1,000 people. Suppressing freedom of expression.	Human Rights Watch (April 23, 2020). The Diplomat.	March 22 to April 14.
10	Turkmenistan	Seizure or restriction of medical personnel cell phones. Forced signing of non-closure forms. Clampdown on freedom of expressions. Ban on wearing of face masks.	Human Rights Watch (April 23, 2020). Rights and Freedoms of Turkmen Citizens.	April 10 to April 23.
11	Morocco	Allegations of human rights abuses. Allegations of police brutality. More than 50,000 arrested.	Moroccoworldnews. Maghreb Arab Press. UN High Commission for Human Rights. BBC, New York Times. Usnews. com	March 15 to March 30.
12	Iran	Non-adherence to WHO advise to release people in crowded prisons due to the coronavirus. Lack in medical supplies and medicines due to US sanctions. Gender-based violence and sexual abuses. Denial of social distancing to prisoners.	The Guardian (Apr 1, 2020). Human Rights Watch (April 6, 2020). Human Rights Watch (March 12, 2020). Al-monitor (Apr 24, 2020).	March 12 to March 30, 2020.

The third stage was literature selection, evaluation and categorizing of the COVID-19 lockdowns/curfews’ reports on human rights abuses, and their correlations to human rights law, statutes and international conventions on human rights. Two criteria were considered in selecting the media reports: first, it must come from a first rated or reputable international or national media, and a reputable national or international human rights organization. Second, it has to include the keywords: “Covid-19 Lockdown”, “Covid-19 Curfews”, “Human Rights Abuses”, “Human Rights Law,” and “International Human Rights’ Conventions,” to justify validity of the literature search.

The fourth stage is the qualitative critical analysis and synthetization of the media reports according to their salience and relationship to study. In doing this, a standardized method of traceable, systematic and reproducible structured content analysis is employed ( Brewerton & Millward, 2001; Seuring & Gold, 2012).

The fifth stage involved the summarization of the findings, the discussion of the findings with emphasis on how it addressed the research questions raised, how it contributed to knowledge and the recommendations for the way forward.

## Results and discussion

Table 1 gives a country by country display of the types of COVID-19 lockdown/curfew human rights’ abuses that occurred in the 12 selected countries within the first two or three weeks of the lockdowns or curfews. These ranged from killings by law enforcement officers, where Nigeria topped the list with 18 persons killed within two weeks of lockdown, followed by Kenya, with 6 deaths. The other rampantly reported human rights’ abuses within the period include: police brutalities, tortures, inhumane and degrading treatment of citizens, arbitrary arrests and detentions, seizure/confiscation of properties, sexual molestations, briberies, denial of social distancing to prisoners, news censorship and suppression of freedom of expression, and denial of social distancing rights to prisoners. Other abuses include tyrannical enforcement of quarantine, and capricious use of force on citizens by police.

Table 2 summarizes the covid-19 lockdown and curfew human rights and freedom abuses that took place in those 12 countries, highlighting the media sources or institutional / organizational sources, the major articles’ titles, their dates and major areas of human rights, freedom or humanitarian rights violations, in reference to human rights’ laws, statutes, international conventions.

**Table 2.** Presentation and analysis of the major media reports’ titles, sources, dates and area of focus on the COVID-19 lockdown and curfew human rights abuses.

S/N	Country	Name of Person, Media or Organisation Reporting	Major Article Title or Related Ones.	Date	Major Area of Focus
1	Nigeria	Amnesty International, NHRC, HRW, Africanews.com, BBC news, Khalid, Ishaq, Aljazeera, Tony Ojukwu. Venturesafrica. Nigeriarights.gov. Saharareporters.	Coronavirus: Security forces kill more Nigerians than Covid-19.	April 14 – 17, 2020	Police kills 18, Covid-19 kills 12 within two weeks.
2	Kenya	United Nations, Aljazeera, Human Rights Watch. Local media.	Kenya authorities investigating 20 cases related to deaths linked to police	April 1-7 2020.	20 deaths linked to police. Brutality.
3	South Africa	Human Rights Watch, South African Human Rights Commission, Local media.	Human Rights Dimensions of COVID-19 Response	March 19 to April 7.	Human rights issues
4	Philippines	United Nations, Aljazeera, HRW, Jamela Alindogan, The Diplomat, Aspinwall Nick,	Police Abuse, Prison Deaths Draw Concern as Philippines Tightens Lockdown Measures	April 28 to 30, 2020	Police Abuses. Prison Deaths.
5	Sri Lanka	HRW, News.Ik, *Meenakshi Ganguly,*	Sri Lanka Uses Pandemic to Curtail Free Expression: Police Ordered to Arrest Critics	April 3, 2020	Freedom of Expression Suppression
6	El Salvador	Human Rights Watch, WOLA, Local media. Miami Herald	Inhumane Prison Lockdown Treatment: President’s Call for Lethal Force, Ignores Basic International Standard	April 15 to 20, 2020	Police brutalities. Arbitrary detentions.
7	Dominican Republic	Daily Mail. *Amnesty International.* Aljazeera.	'Doctor' detained for violating nationwide coronavirus curfew, slapped by police officer	April 20 to 25, 2020	Excessive use of force. Police brutality.
8	Uzbekistan	Human Rights Watch (April 23, 2020). The Diplomat (April 06, 2020).	The Diplomat (April 06, 2020), Uzbekistan Adopts Strict Regulations to Fight COVID-19		Police Abuses. Arbitrary detentions
9	Kyrgyzstan	Human Rights Watch (April 23, 2020). The Diplomat (April 06, 2020)	The Diplomat (April 06, 2020), Inside COVID-19 Quarantine in Kyrgyzstan		
10	Turkmenistan	Human Rights Watch (April 23, 2020). Rights and Freedoms of Turkmen Citizens. Radio Free Europe (April 28, 2020)	Radio Free Europe (April 28, 2020), COVID-19: 'Coronavirus- Free' Turkmenistan Clears Out Quarantine Centers Ahead of WHO		
11	Morocco	Morocco World News. Maghreb Arab Press. UN High Commission for Human Rights.	Reuters (03 May 2020), Morocco rejects accusation of police abuse in enforcing lockdown.		
12	Iran	The Guardian (Apr 1, 2020). Human Rights Watch (April 6, 2020). Human Rights Watch (March 12, 2020).	Al-monitor (Apr 24, 2020), Women, children face increased risk of abuse during Mideast lockdowns.		Gender-based violence and abuses

### Analysis of findings in relation to the research questions

Addressing RQ1: Was there a significant violation of fundamental human rights in the COVID-19 lockdowns and curfews implementations within the first one month?

Data displayed on Table 1 and Table 2 indicate that significant breaches of peoples’ fundamental rights occurred in the covid-19 lockdowns and curfews implementations, especially within the first one month of its implementation in all 12 countries (100%), leading to police-related deaths in two countries and prison-congestion deaths in three countries (33.33%). Nigeria topped the list of countries where citizens lost their lives, with 18 persons killed by law enforcement officers, as opposed to 12 killed by the COVID-19 within the first 14 days of lockdown ( Aljazeera, 2020a; Amnesty International, 2020a; BBC, 2020a; Khalid, 2020). This is followed by Kenya, with six cases of deaths linked to police ( Human Rights Watch, 2020a). The countries that recorded prison deaths due to overcrowding and consequent COVID-19 no-distancing measures were Philippines, El-Salvador and Iran ( Human Rights Watch, 2020b).

Other human rights abuses reported in the 12 countries within the period under review include tortures, inhumane and degrading treatments of people, arbitrary arrests and detentions, seizure/confiscation of properties, sexual molestation, police brutalities and briberies, extortions, shootings, beatings and teargassing people, excessive use of force, gender-based violence, news censorship and suppression of freedom of expression ( Alindogan, 2020; Aljazeera, 2020b; Human Rights Watch, 2020j; The Diplomat, 2020c). These negate the provisions of International instruments like Article 20 of the Universal Declaration on Human Rights, Article 10 of the European Convention on Human Right, (ECHR), Article 9 of the Africa Charter on Human right, all of which guarantee the peoples’ rights to life, liberty, economic and social rights, among others. The violations are also at variance with Article 7, Article 9 (1 & 2), Article 17 and Article 19, International Convention on Civil and Political Rights ( ICCPR, 1976c), that are very essential for rule of law in any constitutional democracy ( Egbewole, 2012). It is also against Article XI of the French Declaration of the Rights of human beings, which all provide for freedom of information and expression.

Addressing RQ2: Did security forces in the countries under review use excessive force to enforce lockdowns and curfew?

A critical analysis of the literature review and data displayed in Table 1 and Table 2 reveal that security forces used tyrannical force to implement lockdowns and curfews in at least 10 out of the 12 countries under review ( Human Rights Watch, 2020e). In Dominican Republic a medical doctor is slapped by police officers ( Dailymail, 2020), news censorship and suppression of free expression were reported in Sri Lanka, the Philippines, Central Asia countries of Uzbekistan, Kyrgyzstan and Turkmenistan ( Ganguly, 2020; Human Rights Watch, 2020e; The Diplomat, 2020a). Out of the 12 countries, it was only Morocco that denied the allegations of police tyrannies against the public when confronted by the mass media, the Human Rights Watch, and other rights organizations ( Reuters, 2020). While President Muhammadu Buhari of Nigeria admitted and apologized to Nigerians regarding police brutalities in the COVID-19 lockdown implementation in the country ( Oloyede, 2020), the President of Kenya, Uhuru Kenyatta, also apologized to his people on the same score ( Human Rights Watch, 2020h).

Addressing RQ3: Were the lockdowns and curfew enforcements significantly lawful, necessary and proportionate?

A doctrinal review of the constitutions of some of the countries under review shows that the lockdowns and curfew enforcements was significantly lawful, to the extent that it was covered in their constitutions. For instance, Section 305(1, 3a, 3b, 3c) of the Nigerian Constitution, 1999, gives government the rights to declare curfew in emergencies or when there is enormous threat to national security or to citizens. This also agrees with the 1985 International Commission of Jurists’ Siracusa Principles. Countries hid under these local and international laws to declare covid-19 lockdowns and curfews, since WHO (2020) declared the pandemic a global emergency. However, fearing that these powers could be abused, a group of United Nations human rights experts, warned countries not to suppress peoples’ rights under the cover of emergencies ( Amnesty International, 2020b; Aljazeera, 2020b; Human Rights Watch, 2020f; OHCHR, 2020; WOLA, 2020).

Addressing RQ4: Were all the citizens of the countries under review guaranteed highest quality health standards possible?

A critical analysis of the literature review and the data in Table 1 and Table 2 reveal that all the citizens of the countries under review were not guaranteed the rights to highest quality health standards possible. For instance, Amnesty International (2020b), reports that Nigerians in their millions living in informal abodes had no access to normal healthcare, hence, highly exposed to COVID-19 infection. Human Rights Watch (2020a), then advised the Nigerian Government to make sure these peoples’ physiological needs are taken care, especially at this time of crisis. Governments also owe it as a duty to ensure that the most vulnerable in society, including persons living with disabilities, and the homeless, are also guaranteed the best chances of survival during emergencies ( Amnesty International, 2020a).

In countries like Philippines, El Salvador, Iran, Turkmenistan, Kyrgyzstan and Uzbekistan, prisoners and people in correctional and detention centres were held in inhuman overcrowded and unhygienic conditions, without any social distancing measures, thus, at risk of contracting COVID-19 ( Human Rights Watch, 2020b; Radio Free Europe, 2020; The Diplomat, 2020a). In Kazakhstan, quarantine measures were also reportedly implemented in arbitrary and harsh manners ( News.IK, 2020). In Sri Lanka, the COVID-19 pandemic was used to enforce press censorship and curtail freedom of expression ( Ganguly, 2020). In Kenya, some of the police that were mandating everyone to wear masks, were themselves not wearing masks and other protective gear ( Human Rights Watch, 2020a), and police threw teargas canister at a man, in Kakamega county, Kenya at midday on April 1, 2020, at the Mumias market, which reportedly killed him ( Human Rights Watch, 2020a).

Over 87million of society’s poorest and most vulnerable people in Nigeria also see their lives and livelihoods being destroyed by the pandemic ( Human Rights Watch, 2020a). COVID-19 lockdown palliatives or social incentives from the Nigerian Government meant for the most vulnerable never reached the majority. This gave rise to a large number of complaints nationwide. According to official reports, about 90 million Nigerians live in extreme poverty (below $2.00 a day) ( World Bank, 2019). President Muhammadu Buhari promised COVID-19 lockdown palliative policies to citizens ( Amnesty International, 2020a), but this still leaves about 87 million vulnerable poor out. These negate part of the provisions of International Bill of Rights, which enshrines the right to health, among other things ( UNHCR, 1976).

Addressing RQ5: Were the lockdowns and curfew enforcements significantly respectful of human life and human dignity?

A critical analysis of the literature review and the data in Table 1 and Table 2 again indicates that the lockdowns and curfew enforcements did not respect human life and human dignity in significant cases. For instance, in Dominican Republic, a medical doctor was beaten, humiliated and detained by police officers for violating nationwide coronavirus curfew ( Daily Mail, 2020). Kyrgyzstan, Kazakhstan, and Uzbekistan also infringed on citizens’ freedom of expression ( Human Rights Watch, 2020g; Radio Free Europe, 2020; The Diplomat, 2020a).

The UN High Commissioner for Human Rights, Michelle Bachelet, advised that effective combat of the COVID-19 pandemic demands countries to ensure that both rich and poor citizens have access to treatment, and shielded from stigma, ( OHCHR, 2020). This is also enshrined in all the International Covenants on peoples’ rights ( ICCPR, 1976d; OHCHR, 2020).

Addressing RQ6: Were the Covid-19 lockdowns/states of emergency significantly used discriminatively against disadvantaged and vulnerable groups?

Our literature review and data in Table 1 and Table 2 show that in its first two weeks, COVID-19 emergencies and lockdowns were used discriminatively against disadvantaged and vulnerable groups in some countries. For instance, police used rubber bullets, tear gas, water bombs and whips to ensure social distancing, especially amongst the poor in South Africa ( Human Rights Watch, 2020a). In Nigeria, it was alleged that those who could not pay bribes to the COVID-19 enforcement officers, mainly poor people, were forced into mandatory quarantine ( Human Rights Watch, 2020a). In El Salvador, disadvantaged people like those of African descent, indigenous peoples, and other groups, were denied access to health care and other protections under the current COVID-19 crisis ( WOLA, 2020). Additionally, many prison detainees, including older people and people with underlying health conditions, were denied their rights by being held in inhuman conditions that threatened their health ( Human Rights Watch, 2020b).

In Iran, the police used violence and humiliation to enforce coronavirus curfews, especially among the poorest and more vulnerable ( Human Rights Watch, 2020i). In other countries, there were increased violence against women during COVID-19 lockdown and emergency. In the Central Asian countries of Kazakhstan, Kyrgyzstan, and Uzbekistan, governments allegedly used COVID-19 restrictions to manhandle journalists, activists and healthcare providers ( Radio Free Europe, 2020; The Diplomat, 2020b). In Turkmenistan, doctors and some other medical personnel were denied access to their cell phones at work ( Radio Free Europe, 2020). The coronavirus outbreak, also witnessed unusual attacks, stigma and discrimination against Asians in many countries ( The Guardian, 2020a).

All these are at variance with the United Nations (1948) Universal Declaration of Human Rights, and other international instruments, charters and covenants on human rights.

### Recommendations

Based on the outcome of this situation analysis, we hereby make the following recommendations:

- Every nation of the world should use social marketing campaigns to mass-educate and enlighten their people more on the realities of the coronavirus, and the consequent dangers of ignoring its preventive measures. When this message is sunk into the peoples’ consciousness, it will make things easier for law enforcement officers enforcing COVID-19 lockdowns and or curfews.
- Social marketing should also be used to organize orientation programs for law enforcement officers enforcing the COVID-19 lockdowns and or curfews on basic human rights and humanitarian rights law, so as to enable them deal with civilians without breaches.
- Human rights and humanitarian rights should characterize the implementation of the COVID-19 lockdowns and or curfews in every country.
- Security forces in every nation should avoid the use of excessive force in enforcing lockdowns and curfews in their countries. No matter how disobedient or stubborn the people might be, the use of persuasive communications and mild force should suffice.
- Every nation must ensure that their lockdown and or curfew are significantly fashioned and enforced in lawful, necessary and proportionate manners, in tandem with international conventions on human
- Every nation must ensure that people in the country are guaranteed the right to the highest quality of health possible, no matter their economic, political or social status.
- Every nation must ensure that their COVID-19 emergency measures are not used to settle scores with political opponents, or against disadvantaged, minorities, or vulnerable people.
- Social marketing campaigns should be used to enlightenment the populace in every nation on the “dos and don’ts” of COVID-19, so that they will see the need to abide by all the advertised preventive measures, and also give their willing cooperation to law enforcement officers.
- Social marketing should be used to enlightenment citizens of every country that to conquer the COVID-19, we must all come together as one and give each supporting hands. In this trying times, everyone in the world must resist the temptation to give in to stigmatization, xenophobia or selfishness. This is the time to be our brother’s keeper much more than ever before, regardless of race, color, creed or religion.

### Suggestions for further studies

Most of the reported human rights’ abuses during the COVID-19 lockdown and curfews occurred mainly in poor and developing countries of the world. Further studies could be directed at determining the remote and immediate causes of this, which will help in forestalling the problem in future in those countries, thus, making the world a better place for all.

## Data availability

All data underlying the results are available as part of the article and no additional source data are required.
