# Supplementary material for: COVID-19 lockdown controls and human rights abuses: the social marketing implications
Source: Emerald Open Res. 2020 Jul 17;2:45. doi: 10.35241/emeraldopenres.13810.1 (PMC8243984; doi:10.35241/emeraldopenres.13810.1)
Supplement: Peer review history

# Peer review: Reviewer response for version 1

**DOI:** 10.21956/emeraldopenres.14888.r27366

**Published:** 2021-02-17

**Authors:** Mario Coccia

**Affiliations:** 1 Research Institute on Sustainable Economic Growth, Collegio Carlo Alberto, CNR - National Research Council of Italy, Turin, Italy

This study endeavors to report human rights abuses that have characterized the COVID-19 controls and lockdown in some countries of the world. 

 The topics of this paper is interesting but the structure and content have to be revised by authors before passing peer review.

 Title can be improved to fit better the content. 

 Introduction has to better clarify the research questions of this study and provide more theoretical background. In particular, authors have to better describe the risk factors of COVID-19, containment measures of lockdown and its socioeconomic effects before to investigate the topics of this study (see suggested readings that have to be read and used in the text).

 I suggest to include research questions in the introduction rather than in the literature review.

 Avoiding and just mentioned in sections, sub-headings that create fragmentation of the paper.

 The sub-heading “origin and spread of COVID-19” I suggest to insert it in Introduction. Now literature review has a lot of subheadings that create confusion,. I suggest to reorganize it, reducing them.

 Table 1 shows some countries under study, I suggest to insert a column to indicate the political system and level of democratization using  Freedom house index to better explain a likely relation with human rights abuse, and also the geoeconomic area of countries under study, such as Iran, Middle East, Unitary Presidential Islamic Republic, Democratization Index Freedom House 15/100 = Not free

 And so on for other countries under study.

 This information is important to support a better discussion also in relation to the length of lockdown applied and likely socioeconomic problems (cf., literature suggested). 

 Methods of this study are not clear. The section of Materials and methods (section 2) has to better clarify the logic of inquiry and how countries have been selected, indicating geoeconomic areas and other sociopolitical characteristics as explained. . 

 Conclusion has to be created and has not be a summary but authors have to focus on manifold limitations of this study and provide suggestions of  health, rule of law, human right  and social policy to avoid future problems in the presence of pandemic crises leading to severe containment measures.

 Overall, then, the paper is interesting but theoretical framework,  study design,  discussion and presentation of results have to be clarified using suggested comments.

 Suggested readings of relevant papers that have to be read and all inserted in the text and references.

 Atalan A. 2020. Is the lockdown important to prevent the COVID-19 pandemic? Effects on psychology, environment and economy-perspective. Annals of medicine and surgery 56, 38-42 https://doi.org/10.1016/j.amsu.2020.06.010

 Coccia M. 2020. An index to quantify environmental risk of exposure to future epidemics of the COVID-19 and similar viral agents: Theory and Practice. Environmental Research, Article number 110155, DOI: 10.1016/j.envres.2020.110155

 Chakraborty I., P. Maity 2020. COVID-19 outbreak: migration, effects on society, global environment and prevention. Sci. Total Environ. 728, https://doi.org/10.1016/j.scitotenv.2020.138882 138882

 Roy S., Dutta R., Ghosh P. 2021. Towards Dynamic lockdown strategies controlling pandemic spread under healthcare resource budget. Applied network science, 6(1), 2. https://doi.org/10.1007/s41109-020-00349-0

 Sabat I., Neuman-Böhme S., Varghese N. E., Barros P. P., Brouwer W., van Exel J., Schreyögg J., Stargardt T. 2020. United but divided: Policy responses and people's perceptions in the EU during the COVID-19 outbreak. Health policy (Amsterdam, Netherlands), 124(9), 909–918 ^1^.

 Coccia M. 2021. The relation between length of lockdown, numbers of infected people and deaths of Covid-19, and economic growth of countries: Lessons learned to cope with future pandemics similar to Covid-19. Science of The Total Environment, Available online 12 February 2021, 145801 ^2^.

 Schlosser, F., Maier, B.F., Jack, O., (...), Zachariae, A., Brockmann, D.    2021. COVID-19 lockdown induces disease-mitigating structural changes in mobility networks. Proceedings of the National Academy of Sciences of the United States of America, 117(52), pp. 32883-32890 ^3^.

 Coccia M. 2020. How (Un)sustainable Environments are Related to the Diffusion of COVID-19: The Relation between Coronavirus Disease 2019, Air Pollution, Wind Resource and Energy. Sustainability 2020, 12, 9709 ^4^. 

 Makhbul, Z.K.M., Rawshdeh, Z.A.  2021. Mental stress post-COVID-19  International Journal of Public Health Science, 10(1), pp. 194-201 ^5^.

Is the work clearly and accurately presented and does it cite the current literature?

Partly

If applicable, is the statistical analysis and its interpretation appropriate?

Not applicable

Are all the source data underlying the results available to ensure full reproducibility?

Partly

Is the study design appropriate and is the work technically sound?

Partly

Are the conclusions drawn adequately supported by the results?

No

Are sufficient details of methods and analysis provided to allow replication by others?

Partly

Reviewer Expertise:

COVID-19, Environmental sciences, Socioeconomic studies of COVID-19, Public policies for COVID-19, Public health

I confirm that I have read this submission and believe that I have an appropriate level of expertise to confirm that it is of an acceptable scientific standard, however I have significant reservations, as outlined above.

## References

[1] : Erratum to "Is the lockdown important to prevent the COVID-19 pandemic? Effects on psychology, environment and economy-perspective" [Ann. Med. Surg. 56 (2020) 38-42]. *Ann Med Surg (Lond)*.56: 10.1016/j.amsu.2020.07.001 217 10.1016/j.amsu.2020.07.001 32562476PMC7293850

[2] : The relation between length of lockdown, numbers of infected people and deaths of Covid-19, and economic growth of countries: Lessons learned to cope with future pandemics similar to Covid-19. *Science of The Total Environment*.2021; 10.1016/j.scitotenv.2021.145801 10.1016/j.scitotenv.2021.145801

[3] : COVID-19 lockdown induces disease-mitigating structural changes in mobility networks. *Proceedings of the National Academy of Sciences*.2020;117(52) : 10.1073/pnas.2012326117 32883-32890 10.1073/pnas.2012326117 PMC777690133273120

[4] : How (Un)sustainable Environments Are Related to the Diffusion of COVID-19: The Relation between Coronavirus Disease 2019, Air Pollution, Wind Resource and Energy. *Sustainability*.2020;12(22) : 10.3390/su12229709 10.3390/su12229709

[5] : Mental stress post-COVID-19. *International Journal of Public Health Science*.2021;10(1) :194-201

# Response

**Published:** 2021-04-04

**Authors:** Ben Odigbo

I wholeheartedly accept the report of this reviewer. It was good work with ample depth. However, we found that not all the countries reviewed are captured under the Freedom house index Freedom house index. So, we will make do with those covered. Thank you and Happy Easter holiday.

 -Dr Ben Odigbo

# Peer review: Reviewer response for version 1

**DOI:** 10.21956/emeraldopenres.14888.r27321

**Published:** 2021-01-25

**Authors:** Patrick Mutahi

**Affiliations:** 1 University of Edinburgh, Edinburgh, United Kingdom

This article is a situation analysis of reported human rights abuses that have characterized the COVID-19 controls and lockdown in some countries of the world. This is as documented by reliable mass media sources, relevant international organizations and human rights non-governmental organizations between January 2020 to April 2020.

 The article is good and can pass peer review. It relies on extensive literature and the arguments made as well as data used are going to be useful for those understanding about covid-19 and effect on human rights.

Is the work clearly and accurately presented and does it cite the current literature?

Yes

If applicable, is the statistical analysis and its interpretation appropriate?

Yes

Are all the source data underlying the results available to ensure full reproducibility?

Yes

Is the study design appropriate and is the work technically sound?

Yes

Are the conclusions drawn adequately supported by the results?

Yes

Are sufficient details of methods and analysis provided to allow replication by others?

Yes

Reviewer Expertise:

policing

I confirm that I have read this submission and believe that I have an appropriate level of expertise to confirm that it is of an acceptable scientific standard.
